# The Design of a Randomized Clinical Trial to Evaluate a Pragmatic and Scalable eHealth Intervention for the Management of Gestational Weight Gain in Low-Income Women: Protocol for the SmartMoms in WIC Trial

**DOI:** 10.2196/18211

**Published:** 2020-09-10

**Authors:** Emily W Flanagan, Abby D Altazan, Natalie R Comardelle, L Anne Gilmore, John W Apolzan, Jessica St. Romain, Julie C Hardee, Renee S Puyau, Christy L Mayet, Robbie A Beyl, S Ariel Barlow, Sarah Surber Bounds, Kelsey N Olson, Betty M Kennedy, Daniel S Hsia, Leanne M Redman

**Affiliations:** 1 Pennington Biomedical Research Center Baton Rouge, LA United States

**Keywords:** mobile health, mobile phone, maternal obesity, gestational weight gain, community health

## Abstract

**Background:**

Less than one-third of women gain an appropriate amount of weight during pregnancy, which can influence the long-term health of both the mother and the child. Economically disadvantaged women are the most vulnerable to maternal obesity, excessive weight gain during pregnancy, and poor birth outcomes. Effective and scalable health care strategies to promote healthy weight gain during pregnancy specifically tailored for these women are lacking.

**Objective:**

This paper presents the design and protocol of a biphasic, community-based eHealth trial, SmartMoms in WIC, to increase the adherence to healthy gestational weight gain (GWG) recommendations in low-income mothers receiving women, infant, and children (WIC) benefits.

**Methods:**

Phase 1 of the trial included using feedback from WIC mothers and staff and participants from 2 community peer advisory groups to adapt an existing eHealth gestational weight management intervention to meet the needs of women receiving WIC benefits. The health curriculum, the format of delivery, and incentive strategies were adapted to be culturally relevant and at an appropriate level of health literacy. Phase 2 included a pragmatic randomized controlled trial across the 9 health care regions in Louisiana with the goal of enrolling 432 women. The SmartMoms in WIC intervention is an intensive 24-week behavioral intervention, which includes nutrition education and exercise strategies, and provides the technology to assist with weight management, delivered through a professionally produced website application.

**Results:**

Phase 1 of this trial was completed in July 2019, and recruitment for phase 2 began immediately thereafter. All data are anticipated to be collected by Spring 2023.

**Conclusions:**

The SmartMoms in WIC curriculum was methodically developed using feedback from community-based peer advisory groups to create a culturally relevant, mobile behavioral intervention for mothers receiving WIC benefits. The randomized clinical trial is underway to test the effectiveness of a sustainable eHealth program on the incidence rates of appropriate GWG. SmartMoms in WIC may be able to offer an innovative, cost-effective, and scalable solution for GWG management in women served by WIC.

**Trial Registration:**

ClinicalTrials.gov NCT04028843; https://clinicaltrials.gov/ct2/show/NCT04028843

**International Registered Report Identifier (IRRID):**

DERR1-10.2196/18211

## Introduction

Pregnancy is a critical time that influences the immediate and long-term health of both the mother and the child. The health and nutritional status of women before pregnancy is arguably the most important factor for long-term health outcomes [1]. Additionally, the nutritional status of women during pregnancy, including gestational weight gain (GWG), influences birth outcomes, maternal and infant health, and long-term risk for chronic disease in mothers [2] and children [3,4]. Fewer than one-third of women gain an appropriate amount of weight throughout pregnancy [5].

Women who are economically disadvantaged are the most vulnerable to inappropriate weight gain during pregnancy [6,7] and poor birth outcomes [8-11]. According to the US Census Bureau in 2015, more than 1 in 8 women and 1 in 4 children live in poverty [12]. The US Department of Agriculture’s Supplemental Nutrition Assistance Program for Women, Infants, and Children (WIC) was established to safeguard the health of low-income pregnant women, infants, and children up to the age of 5 years who are at nutritional risk. Data from the Centers for Disease Control Pregnancy Risk Assessment Monitoring System report that at least 50% of pregnant women supported by WIC have overweight or obesity [13], and only 30% of the women achieve appropriate GWG [14]. The WIC program has an established framework across the United States and provides services to approximately 15% of pregnant women each year [14]. WIC therefore has a unique opportunity to disseminate a weight management program to millions of pregnant women most in need of such programs. Effective and scalable community-based interventions specifically tailored to underserved women are urgently needed.

Pragmatic clinical trials testing scalable, culturally relevant, and appropriately powered interventions aimed at promoting healthy GWG in economically disadvantaged pregnant women are lacking. The SmartMoms in WIC trial will address these gaps by developing and evaluating the effectiveness of an eHealth intervention on the incidence of appropriate GWG in economically disadvantaged mothers receiving WIC benefits. The state-wide randomized controlled trial will test the central hypothesis that relative to WIC participants receiving usual care, participants receiving the newly formed Healthy Beginnings program will have greater adherence to the 2009 Institute of Medicine (IOM; now Academy of Medicine) GWG guidelines and significant improvements in physiological and behavioral factors. The aim of this paper is to describe the development, methodology, and recruitment plan of the SmartMoms in WIC trial.

## Methods

### Study Design

A pilot trial conducted at Pennington Biomedical Research Center (PBRC) in Baton Rouge, Louisiana, showed the efficacy of SmartMoms, an intensive behavioral smartphone intervention to reduce excess GWG in pregnant women with overweight or obesity when compared with delivery via a traditional in-person approach [15]. Notably, as compared with SmartMoms delivered in person, women receiving SmartMoms through a smartphone were more adherent (76.5% vs 60.8%). This trial also showed that a GWG intervention is more cost-effective when delivered through a smartphone device compared with delivery via in-person sessions (smartphone: US $97, SD US $6 vs in person: US $347, SD US $40). In response to the US National Institutes of Health solicitation for research focused on Maternal Nutrition and Pre-pregnancy Obesity: Effect on Mothers, Infants, and Children (PA-18-135), we developed a randomized controlled trial titled: *A pragmatic, scalable e-health intervention for management of gestational weight gain in low-income mothers (SmartMoms in WIC).* The clinical trial is registered at ClinicalTrials.gov (NCT04028843). This work is supported by the National Institutes of Health (R01NR017644) and core support via U54GM104940 and P30DK072476.

**Figure 1:**
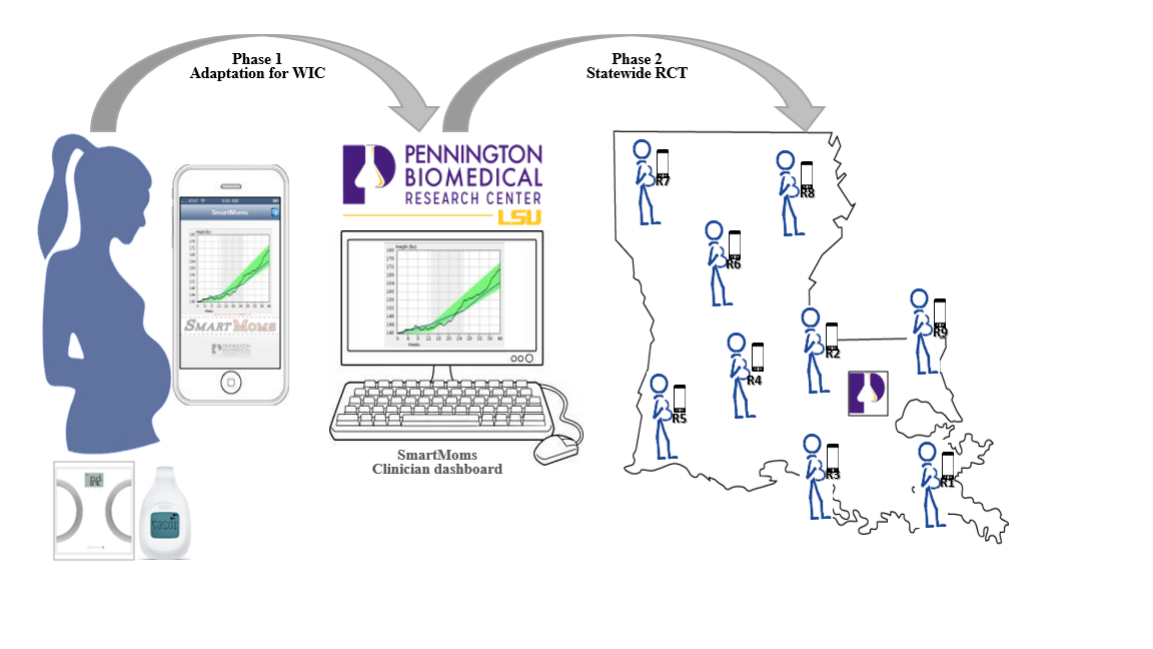
Two phases of the SmartMoms in WIC Trial.

The SmartMoms in WIC study has 2 phases. Phase 1 includes formative research that informs the enhancements made to the previous SmartMoms intervention to better meet the needs of economically disadvantaged women supported by WIC during pregnancy. Phase 2 includes the execution and completion of a state-wide randomized controlled trial. Phases 1 and 2 are depicted in Figure 1. The study is accomplished through close collaboration with the Louisiana Department of Health; Louisiana Special Supplemental Nutrition Program for Women, Infants, and Children; and engagement with 2 community stakeholder groups: Baton Rouge Community Advisory Board of the Louisiana Clinical and Translational Sciences Center (LA CaTS) and a newly formed WIC Mothers’ Advisory Group.

### Phase 1

For phase 1, the specific aim was to use data from formative research surveys of 614 WIC clients and staff from across Louisiana together with semistructured interviews and consultations with a Community Advisory Board and a WIC Mothers’ Advisory Group to adapt SmartMoms for economically disadvantaged women.

#### Adaptation of the SmartMoms Intervention for Low-Income Women

A state-wide qualitative research study (NCT02657577) [16] was conducted as formative research to understand whether WIC would be a suitable venue for SmartMoms and how it should be customized considering the needs and barriers of WIC clients. A random sample of WIC clinics in each of the 9 regions (1-2 clinics per region) for the Louisiana Department of Health was chosen for the surveys. The survey was designed to understand the desire for weight management to be incorporated into WIC services. Results showed that WIC clients and WIC staff at the state, regional, and clinic levels stated that weight management is an important service for WIC. Furthermore, 76% (318/419) of clients expressed a desire to lose weight, and >70% stated they would like to receive weight management services, including cooking classes, support groups, and additional nutrition or lifestyle information through a free app or website. For clients who reported difficulty attending WIC appointments, lack of transportation and time restraints were identified as the primary barriers. In addition, 78% (152/195) of the WIC staff surveyed stated that weight management is needed in WIC. However, WIC staff stated that more time with the client would be needed to adequately implement a weight management program. The survey findings demonstrate that an intervention geared toward weight management in WIC should be accessible through smartphone or internet-based apps as recommended by clients and outside of the WIC appointment to eliminate burden from WIC staff.

Using the results of the WIC survey as a starting point, phase 1 engaged 2 independent stakeholder groups: the Baton Rouge Community Advisory Board of LA CaTS and a newly formed WIC Mothers’ Advisory Group to guide the adaptation of the piloted curriculum of the SmartMoms intervention [15] specific to the wants and needs of economically disadvantaged women. Monthly meetings were held with both groups to produce a curriculum that was more relatable to pregnant women receiving WIC, culturally sensitive, and at an appropriate level of health literacy.

On the basis of the recommendations provided by the WIC Mothers’ Advisory Group, we developed a mechanism for social support, modified the written intervention content to be delivered via short videos, incorporated WIC-approved foods in all dietary materials, produced exercise videos featuring pregnant women, and developed a gamification incentive program. Advisory Group recommendations and the resulting intervention modifications are shown in Table 1.

The Community Advisory Board and the WIC Mothers’ Advisory Group also named the usual care and intervention groups. Usual care participants are enrolled in the *WIC Nutrition* group as all nutrition information is provided through WIC standard care, and the participants receiving the SmartMoms in WIC intervention are enrolled in the *Healthy Beginnings* group.

The advisory boards recommended that women in the usual care group would also benefit from receiving reputable health information throughout pregnancy. Existing health materials unrelated to diet, exercise, and weight gain throughout pregnancy, such as from the American Academy of Pediatrics, are shared with usual care participants once per week for 24 weeks. The weekly curriculum topics of the Healthy Beginnings intervention and usual care groups are shown in Table 2.

**Table 1:** Weekly content for the intervention and control groups.

Advisory Board recommendations	Resulting modification to the Healthy Beginnings interventions
Mechanism for social support	Advisory Group participants wanted a sense of community within the programs. Virtual support groups were created through Facebook. Separate and private groups were created for intervention and usual care groups.
Video-based content	Short, 2 to 4 min videos were professionally produced to deliver intervention lessons to participants.
Relatable recipes	Research dieticians developed 24 recipes that incorporate WIC^a^-eligible foods. Preparation of these recipes are demonstrated in full length (n=10) and fast forward videos (n=14).
Exercises for pregnant women	8 coach-led group exercise classes were produced along with 24 *exercise of the week* videos. All exercise materials are suitable for women to perform safely throughout pregnancy.
Gamification program to incentivize mothers	Mothers wanted to earn points for intervention adherence. The Mommy Market was developed so that participants in the intervention could earn points from demonstrating an understanding of intervention content and redeem points for pregnancy and infant-related products at the Mommy Market.

^a^WIC: women, infants, and children.

**Table 2:** Weekly content for the control and intervention groups.

Week	Healthy Beginnings	Usual care
1	Weight gain in pregnancy	Prenatal vitamin
2	Overcoming barriers to success	Sleep
3	Meal planning and grocery shopping	Preparing for labor and birth
4	Meal prep and healthy cooking	Healthy attachment
5	Portion control and eating patterns	Recommended immunization schedules
6	Behavior chains	Tooth decay in infants
7	Controlling food cues and hunger	How to take a child’s temperature
8	Building social support	Meditation for children
9	Emotional eating	Generosity in children
10	Gestational diabetes	Insect repellant
11	Protein and fat	Conflict resolution
12	Fluids and fiber	Childproofing the home
13	Carbohydrate and sugar	Cell phones
14	Prenatal vitamins	Child safety
15	Social eating	Infant learning
16	Physical activity	Car seats
17	Mindfulness and relaxation	Parenting an infant
18	Managing food cravings and snacking	Postpartum depression
19	Healthy eating on the go	Breastfeeding
20	Stress and sleep	Choosing a pediatrician
21	Postpartum depression	Back to sleep, tummy to play
22	Preparing for labor and birth	Poison safety
23	Breastfeeding	Preparing for baby
24	Optimizing health postpartum	Parenting

### Phase 2

The specific aim of phase 2 is to conduct a randomized controlled trial within Louisiana WIC clinics across the state to test the effectiveness of the Healthy Beginnings intervention on adherence to the 2009 IOM weight gain in pregnancy guidelines. The primary hypothesis is that compared with WIC participants receiving usual weight management care, participants receiving the Healthy Beginnings intervention will have greater adherence to the 2009 IOM weight gain in pregnancy guidelines and significant improvements in physiological and behavioral factors.

The primary outcome is the incidence of adherence to the 2009 IOM guidelines for GWG per week across the second and third trimesters [17]. The starting weight is the participant’s weight measured between 10^0^ and 15^6^ weeks at visit 1. The final pregnancy weight is measured between 35^0^ and 37^6^ weeks at the late pregnancy study visit. The rate of weight gain is equal to the final pregnancy weight minus the starting weight divided by the number of weeks between the 2 measurement dates. According to the 2009 IOM recommendations, the appropriate rate of weight gain in trimesters 2 and 3 is 0.35-0.50, 0.23-0.33, and 0.17-0.27 kilograms per week for women with normal weight, overweight, and obesity, respectively.

The main secondary outcomes (Table 3) include the rate of GWG (kilograms per week), maternal diet, physical activity, quality of life and stress, birth outcomes, WIC food voucher redemption rate, and postpartum weight retention. Subgroup analyses are planned for intervention effects within BMI categories (normal weight, overweight, and obesity) and race or ethnicity, and for women enrolled in Healthy Beginnings, subgroup analyses include intervention adherence.

**Table 3:** SmartMoms in WIC primary and secondary outcomes.

Outcomes	Outcome descriptions
Primary outcome	Incidence of adherence to the 2009 Institute of Medicine’s guidelines for gestational weight gain for normal weight, overweight, and obesity pregnant women
Secondary outcomes	Gestational weight gain per week Maternal diet Physical activity Quality of life and stress Birth outcomes WIC^a^ food voucher redemption rate Postpartum weight retention at 1 year

^a^WIC: women, infants, and children.

### Study Population

The trial aims to enroll 432 pregnant women certified to receive WIC benefits during their current pregnancies, evenly from the 9 WIC regions in Louisiana. Eligibility criteria were established to include a broad range of healthy pregnant women. Inclusion criteria include singleton viable pregnancy, prenatal certification to receive WIC benefits for the current pregnancy (by meeting income requirements mandated by WIC), gestational age <15 weeks at screening, BMI between 18.5 and 40.0 kg/m^2^ using the prenatal WIC certification visit weight, having a smartphone with internet access, and be willing to be identifiable to other study participants in the program because of social interaction via social media. Exclusion criteria include maternal age between <18 and >40 years at screening; current drug, tobacco, or alcohol use; nonpregnancy illness (HIV, cancer, heart disease, or type 1 or type 2 diabetes), hypertension at screening (systolic blood pressure [SBP]>160 mm Hg or diastolic blood pressure [DBP]>110 mm Hg), current mental health or eating disorder, and plans to move out of the state during the study time frame. Furthermore, participants could be excluded after the screening visit if they failed to complete a behavioral run-in task of keeping a food and activity diary with 80% compliance.

### Trial Personnel

The study organization involves 2 teams of personnel: a clinical assessment team and an intervention team. The clinical assessment team consists of trained maternal and infant research specialists employed by Pennington Biomedical. The clinical assessment team staff are present in the WIC clinics to recruit, screen, and consent participants, conduct study visits, and obtain outcome assessment data. The intervention team are trained behavioral health coaches located at the study hub in Baton Rouge, Louisiana. The health coaches randomize participants, manage social support groups, and oversee intervention delivery via the eHealth system [18]. The study investigators and clinical assessment team are blinded to the treatment group assignment until the end of the research trial.

### Recruitment and Randomization

Our randomized controlled trial to test the effectiveness of the Healthy Beginnings intervention began in July 2019. The trial is conducted in partnership with the Louisiana Department of Health and participating WIC clinics throughout the state of Louisiana. The study protocol was approved by the Institutional Review Boards at Pennington Biomedical Research Center (PBRC) and the Louisiana Department of Health.

Participants are recruited from participating WIC clinics dispersed across 9 regions of the Louisiana Department of Health at their prenatal WIC certification visits (Figure 1). The trial received endorsement of the Governor of Louisiana, Secretary for Health, and State WIC Director. The State WIC Director and regional nutritionists helped to identify WIC clinics to collaborate in the trial. A memorandum of understanding was established with each partnering clinic after space and number of monthly WIC pregnancy certifications were confirmed.

Participants are recruited by clinical research staff in person, by referrals from WIC staff, or by posting flyers in client waiting lobbies. Interested participants complete an initial web-based eligibility survey, which collects contact information and asks the participant basic questions regarding their health history and anthropometrics. If the participant satisfies the basic eligibility criteria (ie, gestational age, singleton pregnancy, age, BMI), they are scheduled for a screening visit to obtain written informed consent and determine eligibility to participate in the trial.

Randomization occurs after the first study visit, but before the participant reaches the 17th week of pregnancy to ensure that the program is initiated by the end of the 16th week of pregnancy. The randomization schedule is prepared by a biostatistician and is not revealed to the clinical research team.

### Intervention Goals, Approach, and Rationale

The Healthy Beginnings intervention is a 24-week intensive behavior modification program now customized for pregnant women receiving WIC that seeks to promote healthy GWG through self-monitoring of weight and activity data, automated prescriptive feedback from the mobile app, and personalized feedback from health coaches. All interactions and treatment recommendations between the participant and the health coaches occur remotely via the multimedia functions of a smartphone or computer.

Following randomization, participants in the Healthy Beginnings intervention group are mailed a startup kit, which includes a Fitbit (Fitbit Alta), a cellular-enabled scale (Bodytrace), and instructions on how to use and synchronize the devices and access the Healthy Beginnings app. Participants are instructed to weigh daily, wear the Fitbit throughout their pregnancy, and to synchronize the Fitbit device periodically. These data are automatically and wirelessly transmitted to a clinician and participant dashboard in near real time, where the weight and activity data are automatically plotted on the weight graph and step graph, dynamic charts within the Healthy Beginnings intervention app.

#### Weight Component

**Figure 2:**
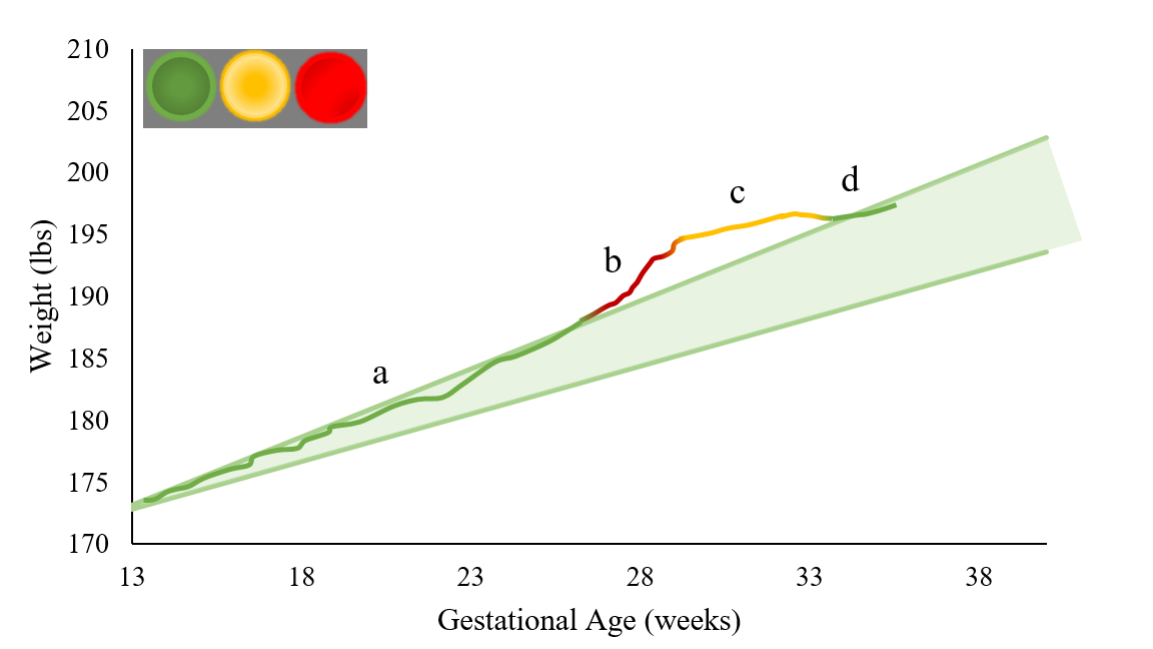
Daily weight charts and visual feedback for a. adherence to recommended rates of weight gain receives an illuminated green light as a visual cue b. above adherence to recommended weight gain receives an illuminated red light as a visual cue c. above recommended rates of weight gain but on a trajectory that is approaching recommended rates of weight gain receives an illuminated yellow light as a visual cue d. weight gain within recommended bounds once again receives an illuminated green light as a visual cue.

Body weight data are displayed on the participants’ personalized weight graph and in relation to their recommended weight change trajectories within the app. An example of the weight graphs is presented in Figure 2. Participants are considered adherent to their personalized weight recommendations if their body weight tracks within the range established by the 2009 IOM guidelines throughout the intervention [19]. Therefore, body weight is used to quantify and depict adherence to the dietary intake goals and to deliver more intensive treatment. Participants are taught that adherence to their weight and activity graphs will promote healthy GWG, but deviation above or below the IOM target guidelines warrants a corresponding change in diet and physical activity. The weight and activity graphs provide participants with automated prescriptive feedback. The feedback includes treatment recommendations bolstered on behavior change theories when participants are out of range (weight or activity level) or congratulatory messages with strategies to maintain success when the weight and activity goals are being met. For added visual feedback, the app automatically generates color-coded visual cues displayed on the graphs indicating whether weights are within the IOM zone of adherence (green), out of the IOM zone (red), or out of the IOM zone but changing at a rate that reflects adherence to the weight gain target (yellow).

When body weight is repeatedly outside the participants’ individualized IOM zone, it serves as an objective indicator to the participant and intervention researchers that more intensive treatment strategies are needed. The intervention team meets weekly to discuss participants with body weight trajectories and adherence to the IOM weight zone. Different treatment strategies to increase the intensity of the weight management program are maintained in a toolbox. Examples of these strategies include increased frequency of contact with the assigned health coach, increased activity or exercise, adoption of a new or revised plan to self-monitor food intake using provided measuring cups to appropriately portion foods and encourage portion-controlled foods (eg, bananas, oranges, carrots, hardboiled eggs, instant oatmeal, yogurt), and advising participants to track foods consumed, which can be done with pen and paper, electronically through free web-based websites and apps, or by sending health coaches photos of their meals. The use of the toolbox strategies is tracked within the app. This toolbox approach is similar to the strategy used in the Comprehensive Assessment of Long-Term Effects of Reducing Intake of Energy (ie, the CALERIE study) [20,21] and Look AHEAD [22], and it provides a systematic and algorithmic method to improve adherence to diet and weight goals.

#### Physical Activity Components

A major focus of the physical activity component is on increasing lifestyle activities, such as taking the stairs, active commuting, and replacing sedentary activities with more active options. Physical activity data are collected and tracked via Fitbit and plotted on the SmartSteps graph within the app. Similar to weight graphs, automated personalized messages are deployed with added contact from intervention researchers, as needed. Both aerobic and resistance-based physical activity is encouraged each week through the *exercise of the week* and group exercise class videos.

#### Diet Component

Participants are encouraged to follow a healthy eating pattern as described in the 2015-2020 USDA Dietary Guidelines for Americans and use MyPlate [23]. The behavioral intervention and developed recipes encourage the intake of nutrient-dense foods including whole grains, fruits, lean protein sources, nuts, and seeds. The intervention also offers strategies to reduce or eliminate sugary drinks, desserts, and fried and rich foods and teaches MyPlate portion sizes. Participants are not required to track food intake on a daily basis; however, intervention researchers can request participants to use their smartphone camera to capture food images to be stored in the toolbox to determine the types of foods they are eating and assist with personalized coaching on the basis of their actual food intake patterns.

#### Intervention Details

In addition, following feedback and recommendations from the advisory boards during phase 1, the newly adapted Healthy Beginnings intervention includes a private Facebook group, a gamification incentive program, and >100 informational, exercise, and cooking videos.

#### Videos

There are 55 professionally produced videos to deliver intervention content and foster behavior change. Each weekly topic is broken down into 2 or 3 short lessons with supporting videos. The intervention content (Table 1) is new each week and is sent to the participant via an automated email. The email contains a summary description of the weekly topic and a website link to the short video hosted on a web-based platform (YouTube). The videos are designed to explain the topic in a simple and straightforward manner. Videos are 2- to 4-minutes long, feature pregnant women, and strongly emphasize the health benefits of the desired behavior change with regard to the health and development of their baby. In addition to the informational videos, research dietitians developed >24 recipes that incorporate WIC eligible foods and are modest in cost. The preparation of these recipes was demonstrated in either full length (n=10) or fast forward videos (n=14). Videos included variations in recipes (eg, incorporating different protein options, including vegetarian when available) and some included side dishes. Eight coach-led group exercise classes were also professionally produced along with 24 *exercise of the week* videos. All exercise materials were suitable for women to perform safely throughout pregnancy. The guided recipe and exercise videos are posted weekly and on a 24-week rotation to the Healthy Beginnings social media page.

#### Social Support

Participants in usual care and the Healthy Beginnings interventions are added to separate and private Facebook groups, where interventionists lead conversations and encourage participant engagement. The intervention manager has a PhD in human clinical nutrition and holds the American College of Sports Medicine–Certified Exercise Physiologist certification. The intervention team (health coaches) hold bachelors or master’s degrees in Kinesiology (fitness and human performance) or in Human Nutrition, a registered dietitian nutritionist, or certified diabetes educator certifications. The group is also monitored by an independent monitor in the Institutional Communications Department to ensure that the interactions between the interventionists and the participants remain aligned with the study objectives. The social media group of Healthy Beginnings is the platform where recipe and exercise videos are posted. The *secret* function is enabled to conceal groups and member identities from outsiders. Membership in the group occurs on a rolling basis. Once participants are enrolled into the trial, they remain in the group following birth, unless they choose to remove themselves. Thus, all women randomized can remain in their respective private Facebook groups until 1 year postpartum.

#### Gamification

To boost participant engagement with the intervention, we developed a gamification component. Participants earn points for engaging with health coaches, completing intervention tasks, and demonstrating behavior change. Accrued points are redeemable for pregnancy and infant-related products recommended by the advisory groups in phase 1, including diapers, bathing products, delivery robes, diaper bags, portable cribs, and strollers. Points can be redeemed for these items once per month when the *Mommy Market* store is opened. A variety of items are offered within 3-point categories of increasing value. The points required for any item are based on the goal of 70% adherence to the intervention, as this level of adherence was required to produce significant weight management results in our past eHealth study in WIC mothers [24].

### Usual Care Group Strategy

Analogous with previous trials embedded in the WIC program [24,25], participants assigned to the usual care group will receive all aspects of the standard WIC program, including standard prenatal weight management advice provided by WIC staff plus a brief phone conversation to orient and bond the participant to the study. Weight management from WIC is administered through a pregnancy health pamphlet that encourages use of the American College of Obstetrics and Gynecology weight gain recommendations. To boost retention, the usual care participants also receive weekly health information unrelated to diet, physical activity, or weight management, delivered via a private Facebook group that is separate from the intervention group. Women in the usual care group receive monthly phone calls to keep participants engaged in the study.

### Study Retention

For convenience, study clinic assessment visits are typically scheduled to correspond with each participant’s scheduled WIC appointment. Additionally, incentives are provided to all participants on an intermittent schedule to promote engagement and improve retention of both the intervention and usual care groups. All participants are compensated US $25 at the completion of each study visit. Incentive items provided at clinic visits include measuring cups and spoons, mixing bowls, water bottles, yoga mats, hand weights and resistance bands, and a tote bag. Participants in both groups are provided the same incentive items as the clinical assessors who are blinded to the intervention assignment distribute incentives at clinic visits. Prenatal vitamins are also offered to all participants throughout pregnancy and are refilled at each prenatal clinic visit. Although incentive items are provided to participants of both groups, the schedule of incentive administration is strategically paired with intervention content. Participants in the Healthy Beginnings intervention have the ability to receive additional incentive items through the gamification component *Mommy Market*, as explained previously. Items earned through the *Mommy Market* are mailed to participants to not reveal blinding to clinic assessors.

### Intervention Adherence

Adherence to the Healthy Beginnings intervention is based upon participant interaction with the weekly intervention curriculum. To receive adherence points, the participants must comment on the video or have a discussion with their health coach (via text, email, phone call, or video call) and demonstrate a basic understanding of the material. Low adherence is defined as ≤40% of session interaction, medium adherence is reaching 40.1%-70% of session interaction, and high adherence is reaching >70% of session interaction.

There are additional components of the intervention, including daily weighing and step counting, which may signify intervention adherence. However, we expect these intervention outcomes to trend similarly with the session interaction and have chosen the weekly study curriculum as the main adherence metric of the intervention as it encompasses these additional components.

### Safety Monitoring

All adverse events, serious adverse events, and safety alerts are reviewed by the study medical investigator (DH) on an ongoing basis. Any significant health problems identified during the study will be referred to the participant’s usual source of medical care, with her permission. Adverse events are defined as any untoward medical occurrence that may or may not be associated with participation in the study. A serious adverse event is an untoward medical occurrence, whether associated with study participation or not, which results in one of the following: death, life-threatening event, hospitalization, preterm delivery before 32 weeks of gestation, disability or permanent damage, or medication intervention to prevent permanent impairment or damage. A contraindication to physical activity during pregnancy [19] results in modification of the Healthy Beginnings intervention.

Additional safety alerts that require medical monitoring are high blood pressure (SBP≥160 mm Hg and/or DBP≥110 mm Hg) and weight loss during pregnancy. Weight loss safety alerts are BMI specific and compared with the first weight assessed in the clinic (normal BMI at enrollment: any weight loss; overweight at enrollment: a weight loss 4%; obesity at enrollment: weight loss 6%).

### Outcome Measures

Through our previous community-based trials [24], we learned that it was critical to embed clinical assessment researchers into WIC clinics as opposed to relying on WIC staff for trial implementation. Study assessments are therefore conducted at the WIC clinic by certified clinical researchers. After the screening visit, there are 6 study visits throughout the trial: 3 visits during pregnancy and 3 visits during the first year postpartum. Pregnancy outcomes are obtained before randomization at 10^0^ to 15^6^ weeks, approximately 12 weeks after enrollment at 24^0^ to 27^6^ weeks of gestation, and close to term (35^0^ to 37^6^ weeks). Women are followed up for 12 months after delivery, with 3 study visits occurring at 2^0^ to 6^6^ weeks, 6 months (22^0^ to 25^6^ weeks), and 12 months (48^0^ to 56^6^ weeks) postpartum. The assessment schedule is presented in Table 4.

**Table 4:** Assessment schedule.

Assessments	Screening	Early pregnancy	Mid pregnancy	Late pregnancy	1 month postpartum	6 months postpartum	1 year postpartum
Height	✓ ^a^	—^b^	—	—	—	—	—
Weight	✓	✓	✓	✓	✓	✓	✓
Percentage fat by BIA^c^	✓	✓	✓	✓	✓	✓	✓
Behavioral run-in	✓	—	—	—	—	—	—
Skinfold thickness	—	✓	✓	✓	✓	✓	✓
Waist and hip circumferences	—	✓	✓	✓	✓	✓	✓
Blood pressure	✓	✓	✓	✓	✓	✓	✓
Questionnaires	—	✓	—	✓	✓	✓	✓
Diet recall	—	✓	—	✓	—	✓	✓
Accelerometry	—	✓	—	✓	—	✓	✓
WIC^d^ chart data abstraction	—	—	—	✓	—	—	✓
Birth certificate data abstraction	—	—	—	—	✓	—	—

^a^Assessment performed during this visit.

^b^Assessment not applicable or not performed during this visit.

^c^BIA: bioelectric impedance scale.

^d^WIC: women, infants, and children.

#### Maternal Outcomes and Measurements

Outcome assessments include a nonfasting measurement of height (screening visit only), weight, blood pressure (measured in duplicate after a 5-min rest), percent fat by bioelectrical impedance, skinfold thickness, hip and waist circumference, physical activity and sleep over 5 to 7 days using the Actigraph GTX3, dietary intake by 24-hour recall (National Institutes of Health, National Cancer Institute ASA-24), and evaluation of depression and anxiety (Depression Anxiety Stress Scales-21) [26], quality of life (Quality of Life Inventory) [27], household chaos (Household Chaos Questionnaire) [28], and infant feeding (Infant Feeding Styles Questionnaire) [29]. Upon delivery, the WIC chart and birth certificate are abstracted to provide data on prenatal, birth, and infant outcomes. To minimize missing data, home visits are permitted when a participant is unable to get to her WIC clinic (ie, bedrest).

#### Offspring Outcomes

The WIC chart of the infant will be abstracted to provide information on delivery outcomes (ie, adverse events, delivery complications, and route) and infant anthropometrics at birth. This will allow us to determine large or small for gestational age infants. As the mother will be followed until her infant’s first birthday, the WIC chart will also have infant hemoglobin and weight and height measured at infancy and at 1 year.

### Data Management and Analysis Plan

#### Data Storage

All data are entered into REDCap, a centralized data management system that allows for real-time, web-based data entry for community-based studies. Diet recall and Actigraph data are saved and stored on a centralized server that is only accessible to the study team.

#### Sample Size and Power Calculations

The sample size estimates assume β=0.8 and α=0.05 to detect the specified increases in the incidence rate of appropriate GWG. The sample estimate is inflated to allow 15% loss of data and maintain the desired power. The loss to follow-up accounts for participants who may drop out of the study and those who may be required to discontinue the intervention because of contraindications or safety alerts. A 15% discontinuation rate has previously been observed in our pregnancy trials [30]. The incidence rates of appropriate GWG for usual care were derived from women receiving WIC benefits in the 2013 Louisiana Pregnancy Risk Assessment Monitoring System data. The estimated effect size for SmartMoms in WIC is based on our pilot trial data where the incidence of appropriate GWG for women with overweight was 12.3% for usual care versus 43.8% for SmartMoms; and for women with obesity was 0% for usual care and 42.9% for SmartMoms. However, as the pilot trial was not in an exclusive underserved population, we conservatively estimate that the Healthy Beginnings intervention will increase the incidence of appropriate GWG by a minimum of 14% across each BMI category. The resulting overall incidence rate would be similar to the observation in our previous trial [15].

In addition to the overall change in incidence rates, this study is also powered to detect BMI group changes in incidence rates. Assuming an overall average change in incidence rates of 14%, 122 participants in each BMI group (accounting for 15% attrition) are sufficient to detect an incidence rate that is at least 20% different between BMI groups.

We will also investigate the rate of GWG per week. According to the 2009 IOM guidelines, each BMI category is allocated a different rate of GWG per week [31]. We will investigate the deviation between the observed rates of GWG and rates deemed appropriate by the 2009 IOM. On the basis of our pilot data, women with overweight and obesity in the SmartMoms group had similar deviation rates (gaining an additional 0.06 kg per week above the recommended amount), whereas the usual care group had a much higher rate of weekly GWG. With our planned sample size of 432 participants, we have >80% power to detect changes as small as 0.12 kg per week between the Healthy Beginnings intervention and usual care and within each BMI category, and >99% power to show overall differences in rates of weekly GWG.

#### Analytical Plan

Statistical analyses will be completed using SAS/STAT software, version 9.4, of the SAS System for Windows by a PhD-level biostatistician. All tests will be performed with significance level α=.05 and using an intent-to-treat analysis. Outcomes will be assessed for normality (where appropriate) using the Shapiro-Wilk test. If transformed data are still not normally distributed, then nonparametric analyses will be conducted on these outcomes. Least-angle regression (LARS) will be used for the covariate selection methodology. Covariates will include, but may not be limited to, the infant’s sex, gestational age at delivery as well as and the maternal age, weight at screening, parity, race or ethnicity, and gestational diabetes status. Treatment and BMI categories will be included in all models. Intent-to-treat analysis will be the primary analysis type. Both completers and multiple imputation (Markov chain Monte Carlo method, preferred) may also be performed if there is a large amount (>10%) of missing data. However, we will extract the mothers’ WIC record and therefore for participants who fail to return to the clinic, weight measurements will be acquired from the planned chart abstraction. This should greatly decrease the amount of missing data from loss to follow-up.

For the incidence rate of appropriate GWG, initial results will be expressed contingency tables with incidence rates and either treatment or BMI category. Tests for associations between these variables will be based on the Pearson chi-squared statistic. Further results will be based on a generalized linear mixed effect model with a binary distribution and a log odd link function modeling the incidence rates. The model will use random effects to account for a site effect and within-subject correlation over time. Results from this model will be reported as odds ratios of least square means, with *P* values based on z tests. Two different sets of models will be used. The first set of models will contain only treatment and BMI categories, whereas the second will also contain the covariates based on LARS. Pairwise post hoc comparisons of the category variables will be corrected for multiple comparisons when necessary.

For the key secondary outcome of weekly GWG rate, overall deviation rates will be reported using a linear effect mixed model, using random effects to account for site and within-subject correlation. The response is the deviation of the weekly GWG rates from the IOM guideline rates. Models will be reported using both the simple model with treatment and BMI categories and with the covariates selected by LARS. Adjusted means of the deviations will be obtained using the least squares means. A two-sample *t* test will be used to compare adjusted mean deviations. For other secondary outcomes, each physiological and behavioral factor will be investigated independently from each other. Linear mixed effect models will be used to test differences between the treatment groups with least square means. The response for each of these models will include both repeated measures and percent change from baseline approaches.

## Results

Phase 1 of this trial was completed in July 2019. The delivered outcomes of phase 1 are the produced Healthy Beginnings intervention curriculum, including development of the gamification component of the intervention and adaptability of the intervention to ensure cultural sensitivities and an appropriate level of health literacy. The produced Healthy Beginnings intervention curriculum is being used in the randomized controlled trial in phase 2. The randomized controlled trial is ongoing, and all data are anticipated to be collected by spring 2023. The final sample of 432 enrolled women is expected to be diverse in BMI and geographic region.

## Discussion

With the recent identification that pregnancy may program the future health of mothers and babies, there has been heightened interest in developing programs in pregnancy that foster appropriate GWG. There is a growing list of trials showing that behavioral interventions focused on diet and physical activity modification can reduce excess GWG in pregnant women [15,32-34]. The next phase of research calls for the translation of effective approaches to community-based programs with the overarching goal to offer more widespread benefit to mothers and their children. Implementation research requires consumer and stakeholder engagement not only to ensure effective translation of the program into the community but also in the design of pragmatic trials in which the community-based eHealth program can be properly evaluated.

The SmartMoms in WIC project is unique in that it is a biphasic clinical trial. First, phase 1 of the project utilizes a formative research model to incorporate feedback from key stakeholder groups in the adaptation of the intervention for economically disadvantaged pregnant women and development of the new curriculum with increased specificity. Second, the newly adapted eHealth behavioral intervention, Healthy Beginnings, is studied in a pragmatic, state-wide randomized controlled trial to test its effect on increasing the incidence of appropriate GWG for women enrolled in WIC across Louisiana.

In our pilot trial, we demonstrated that our SmartMoms intervention was more cost-effective than the same intervention delivered in person. Thus, successful implementation of the new Healthy Beginnings intervention would provide a scalable, personalized, and cost-effective intervention to promote adequate GWG and ultimately to minimize adverse pregnancy, infant, and postpartum outcomes. It is estimated that 77% of adults in the United States own a smartphone [35], and in low-income households, families are more likely to rely solely on mobile devices for internet access [36]. With smartphones, individuals are *mobile* and less reliant on computers for internet access. Furthermore, many insurance programs offer smartphones for pregnant women throughout their pregnancy. The WIC program already offers several smartphone apps [37], and WIC could choose to adopt this intervention framework for use by their pregnant clients. Therefore, if the Healthy Beginnings intervention proves effective in increasing the rates of healthy GWG, the packaging of health information via a smartphone app available to all pregnant women utilizing WIC services makes widespread implementation and distribution feasible and with little burden from WIC staff, which is commonly acknowledged as a roadblock for the implementation of longstanding programs. Furthermore, as the intervention is delivered remotely, the Healthy Beginnings intervention is scalable and has the potential to reach WIC clinics beyond the state of Louisiana.

SmartMoms in WIC has several strengths that distinguish the trial and promote an advance in the field. First, formative research to adapt the curriculum for the target population through 2 independent community advisory groups ensures that health information is relevant and engaging when maintaining value systems and cultural beliefs. Second, the trial is being conducted statewide and aims to evenly enroll women across the 9 regions of the Louisiana Department of Health. This distribution avoids confounding effects of geographical location or WIC clinic staff influence. Third, near real-time participant feedback from the smartphone allows for immediate participant engagement in weight and physical activity levels, which allows participants to make necessary behavior changes independently. However, if corrections are not met, intervention staff will coach the participant through difficulties, providing added support to assist in meeting weight gain goals. Flexibility in participant clinic visits provides the greatest chance of success in obtaining primary outcome measures. If participants move outside the area within the study timeframe but remain within the state of Louisiana, study outcomes can be obtained at the participant’s new WIC clinic. Understanding that transportation is often a barrier for low-income women to attend clinic visits, home visitation is permitted by clinic assessment researchers if a participant’s transportation situation changes throughout the study timeframe. Finally, as smartphone use is already highly prevalent in low-income populations, eHealth interventions can overturn many of the reported barriers for intervention adherence and completion.

Although the Healthy Beginnings intervention is designed with widespread implementation in mind, the current randomized controlled trial does not yet test implementation and integration of the program into WIC. Although the partnership with WIC is an essential component for trial success, it is important to recognize that the trial is led by an experienced research team independent of WIC. Therefore, if the study demonstrates the effectiveness of the intervention for increasing compliance with the IOM recommendations for weight gain in pregnancy, the next step will be to test implementation within the WIC clinics. A limitation of pragmatic trials involving community stakeholders is the possible disconnect between the stakeholder groups and funding bodies. For example, the WIC Mothers’ Advisory Group requested that the outcomes of the trial include health markers other than simply weight gain (eg, glucose and lipids obtained through capillary blood). The mothers explained that understanding the full health benefit of adhering to a lifestyle program in pregnancy would be more informative for their future participation. Interestingly, the scientific review panel was less enthusiastic about including such measures. It is important to note that there may be a disconnect between the crosstalk of community stakeholders and scientific reviewers in the value of additional health information, which can pose a challenge when executing community-based research.

### Conclusions

The status of maternal health during pregnancy has lifelong consequences for women and children and is therefore a public health problem. Furthermore, inappropriate weight gain in pregnancy is a significant health issue, particularly in low-income women, increasing the probability of adverse pregnancy outcomes. The original SmartMoms curriculum was methodically adapted using feedback from community-based peer advisory groups to create a culturally relevant mobile behavioral intervention. The new Healthy Beginnings intervention promotes healthy eating behaviors and physical activity patterns in economically disadvantaged pregnant women through education, immediate participant feedback, and coaching from trained intervention staff. If Healthy Beginnings is efficacious when embedded in the Louisiana WIC program, it will provide the first indication that WIC services could be efficiently expanded to meet an important public health need and serve as a conduit for delivering an eHealth intervention to pregnant women across the country. Furthermore, given the long-term follow-up of children in the WIC program, this study also provides a framework for future investigations into the effects of pregnancy interventions on health outcomes in children.

## Abbreviations

DBP: diastolic blood pressure
GWG: gestational weight gain
IOM: Institute of Medicine
LA CaTS: Louisiana Clinical and Translational Sciences Center
LARS: least-angle regression
PBRC: Pennington Biomedical Research Center
SBP: systolic blood pressure
WIC: women, infants, and children
